# Intensity and directionality of bat echolocation signals

**DOI:** 10.3389/fphys.2013.00089

**Published:** 2013-04-25

**Authors:** Lasse Jakobsen, Signe Brinkløv, Annemarie Surlykke

**Affiliations:** ^1^Institute of Biology, University of Southern DenmarkOdense, Denmark; ^2^Department of Biology, Western UniversityLondon, ON, Canada

**Keywords:** intensity, directionality, beam shape, bat, echolocation, biosonar

## Abstract

The paper reviews current knowledge of intensity and directionality of bat echolocation signals. Recent studies have revealed that echolocating bats can be much louder than previously believed. Bats previously dubbed “whispering” can emit calls with source levels up to 110 dB SPL at 10 cm and the louder open space hunting bats have been recorded at above 135 dB SPL. This implies that maximum emitted intensities are generally 30 dB or more above initial estimates. Bats' dynamic control of acoustic features also includes the intensity and directionality of their sonar calls. Aerial hawking bats will increase signal directionality in the field along with intensity thus increasing sonar range. During the last phase of prey pursuit, vespertilionid bats broaden their echolocation beam considerably, probably to counter evasive maneuvers of eared prey. We highlight how multiple call parameters (frequency, duration, intensity, and directionality of echolocation signals) in unison define the search volume probed by bats and in turn how bats perceive their surroundings. Small changes to individual parameters can, in combination, drastically change the bat's perception, facilitating successful navigation and food acquisition across a vast range of ecological niches. To better understand the function of echolocation in the natural habitat it is critical to determine multiple acoustic features of the echolocation calls. The combined (interactive) effects, not only of frequency and time parameters, but also of intensity and directionality, define the bat's view of its acoustic scene.

## Introduction

The evolutionary success of bats is accredited to their ability, as the only mammals, to fly and navigate in darkness by echolocation, thus filling a niche exploited by few other predators. Over 90% of all bat species use echolocation to localize obstacles in their environment by comparing their own high frequency sound pulses with returning echoes (Griffin, 1958). The ability to localize and identify objects without the use of vision allows bats to forage for airborne nocturnal insects, but also for a diverse range of other food types including motionless perched prey or non-animal food items (Schnitzler and Kalko, 2001; Brinkløv et al., 2011; Geipel et al., 2013).

The agility and precision with which bats navigate and forage in total darkness, is in large part due to the accuracy and flexibility of their echolocation system. The echolocation clicks of the few echolocating Pteropodidae (*Rousettus*) are fundamentally different from the echolocation sounds produced in the larynx that we focus on here, and thus not part of this review. Many studies have shown that bats adapt their echolocation calls to a variety of conditions, changing duration and bandwidth of each call and the rate at which calls are emitted in response to changing perceptual demands (Griffin et al., 1960; Schnitzler and Kalko, 2001). In recent years the intensity and directionality of echolocation signals has received increasing research attention and it is becoming evident that these parameters also play a major role in how bats successfully navigate and forage. To perceive an object in its surroundings, a bat must ensonify the object with enough energy to return an audible echo. Hence, the intensity and duration of the emitted signal act together to determine how far away a bat can echolocate an object. Equally important is signal directionality. Bat echolocation calls are directional, i.e., more call energy is focused in the forward direction than to the sides (Simmons, 1969; Shimozawa et al., 1974; Mogensen and Møhl, 1979; Hartley and Suthers, 1987, 1989; Henze and O'Neill, 1991). An object detectable at 2 m directly in front of the bat may not be detected if it is located at the same distance but off to the side. Consequently, at any given echolocation frequency and duration, it is the combination of signal intensity and signal directionality that defines the search volume, i.e., the volume in space where the bat can detect an object.

The aim of this review is to summarize current knowledge about intensity and directionality of bat echolocation calls, and show how both are adapted to habitat and behavioral context. Finally, we discuss the importance of active motor-control to dynamically adjust both signal intensity and directionality to solve the different tasks faced by echolocating bats.

## Intensity

Call intensity is a main determinant of echolocation range, i.e., the distance from a bat where objects, such as obstacles and food, reflect echoes intense enough for detection. The more intense the call, the further sound travels from the bat and the larger the echolocation range. Emitted intensities (source level) of bat echolocation signals are referenced to a standard distance of 10 cm from the bat's mouth. Thus, when recording bats at a distance, one must add the transmission loss due to geometric spreading [20 × log_10_(R)] and frequency-dependent atmospheric attenuation (ANSI, 1995) over the distance from the bat to the microphone.

When Griffin first investigated how loud bats call, he found that insectivorous bats flying in open space, e.g., aerial hawking vespertilionids, called at around 110 dB SPL (Sound Pressure Level; re. 20 μPa at 0.1 m) and closed-space gleaners operating in or near vegetation, like the phyllostomid *Carollia perspicillata*, called at around 70 dB SPL (Griffin, 1958). Consequently, Griffin divided bats into two groups, the loud insectivorous bats, and the “whispering” gleaning bats. Recordings from the field have since shown that bats are orders of magnitude louder than what Griffin measured, and the border between loud and whispering is much blurrier than initially believed. Open-space insectivorous bats emit calls up to, and beyond, 140 dB SPL (Holderied et al., 2005; Surlykke and Kalko, 2008). Remarkably, even “whispering” bats are capable of emitting calls up to 110 dB SPL (Brinkløv et al., 2009). This means that, while echolocation in air is still a relatively short-range system, its range is considerably larger than first assumed.

**Figure 1:**
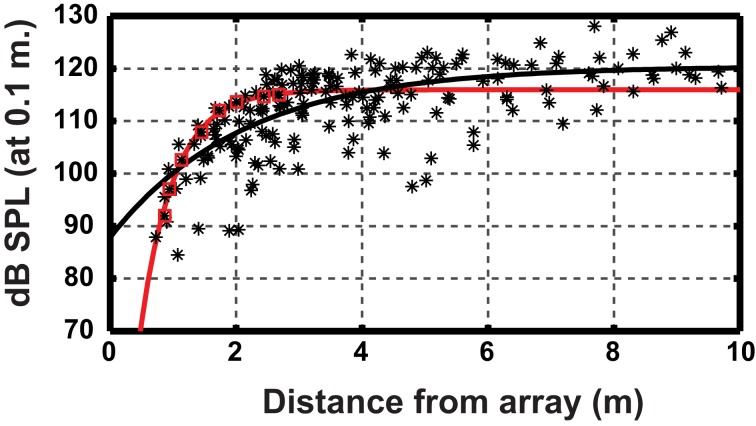
Relationship between source level and distance to the microphone array for *Myotis daubentonii*. Datapoints (^*^) from 14 approaches. The black line is the least-squares fitted exponential function to the entire data set (for details see Nørum et al., 2012). Red squares highlight a single approach and the red line is the fitted function using only these points, showing a much steeper slope. The figure illustrates that estimating the slope using a large data set, comprising many individual steep slopes with different onsets, can yield an artificially shallow slope as compared to individual approaches. Data points from Nørum et al., (2012).

The huge difference between the values for signal intensity obtained by Griffin and more recent measurements illustrates the great flexibility of the echolocation system. Bats dynamically adjust signal intensity to changes in their environment and the task at hand, lowering the output as they approach objects such as prey or vegetation. The dynamic range, or the difference between the loudest and the quietest calls emitted by individual bats is in the order of at least 30–40 dB for most species. When object detection occurs at long range or under predictable lab conditions most studies report a reduction in output level of around 6 dB for every halving of distance to the target (Hartley, 1992b; Hiryu et al., 2007, 2008; Brinkløv et al., 2010; Koblitz et al., 2010, 2011; Nørum et al., 2012). If the object reflects impinging sound like a point target, the echo level at the bats ears would increase by 12 dB per halving of distance if the bat emitted a constant source level. Thus, the consequence would be an enormous increase in echo level through a pursuit, e.g., +80 dB from detection at 5 m to capture at 5 cm, likely to overload central auditory processing. The output reduction of 6 dB per halving of distance removes half of the echo increase such that the sound pressure at the bat's ear increases by only 6 dB per halving of distance. Further, psychophysical experiments have shown that sensitivity on the receiver side is not constant, but decreases by the remaining 6 dB for each halving of distance probably due to contraction of the bats middle-ear muscles (Suga and Jen, 1975). Hence, in a predictable situation the combined adjustment of output and input results in echoes perceived at a relatively stable intensity (Henson, 1965; Suga and Jen, 1975; Hartley, 1992a). Data from more unpredictable situations in the natural environment have also indicated a 6 dB reduction in signal output intensity per distance halved. New data, however, show that the reduction in intensity for individual approaches is mostly much steeper in the wild, up to as much as 30 dB per halving of distance, with considerable variation. The relatively shallow slopes reported from other field studies are probably the result of pooling multiple sequences with steep slopes but initiated at different distances (Figure 1, Nørum et al., 2012). The results suggest that sudden detection of prey or obstacles at close range may prompt an initial dramatic intensity reduction. Curiously, for bats landing on an extended surface, the reduction in output intensity is likewise within the 6 dB per halving of distance range (Koblitz et al., 2011). If the sensitivity on the receiver side changes as well, this results in a gradual decrease in perceived echo strength as the bat approaches the surface.

There may be a less clear-cut separation between loud and whispering bats than previously assumed, but it is still evident that bats flying close to or within dense vegetation are considerably less intense than bats flying in open space. This is true for species that differ in overall habitat use, but also for individual bats switching between habitats with varying degrees of clutter. Under field conditions, the trawling insectivorous phyllostomid *Macrophyllum macrophyllum* lowers mean signal intensity from 111 dB SPL in open space to 105 dB SPL in semi-cluttered space. Signal intensity is further reduced to 100 dB SPL when *M. macrophyllum* navigates a small flight room, demonstrating an obvious dynamic adjustment of output intensity in response to varying degrees of habitat clutter (Brinkløv et al., 2010).

The adjustment of signal intensity in *M. macrophyllum* occurs in parallel with changes in signal duration and peak frequency. Open space calls are not only louder, but also longer and with lower peak frequency than those emitted in semi- or densely cluttered conditions. These changes all contribute to an increase in sonar range in open space. The increased duration increases the signal energy and the lower frequency reduces the effects of atmospheric attenuation. Attenuation of sound in air increases drastically with frequency (Lawrence and Simmons, 1982; ANSI, 1995) which presumably represents a major constraint for echolocating bats resulting in a trade-off between sonar range on one hand and resolution and localization on the other (Kalko and Schnitzler, 1993). The low intensities and high frequencies emitted by most gleaners in clutter likely indicate that sonar range is not an issue. Thus, the low intensities serve to prevent self-deafening and the high frequencies serve to increase resolution and localization (Kalko and Schnitzler, 1993).

The use of high frequencies also increases clutter rejection along the acoustic axis when the prey is closer to the bat than the clutter. This is because the increased atmospheric attenuation at higher frequencies will generate a relatively weaker echo the further away an object is. An increase in emitted frequency from 45 to 90 kHz increases atmospheric attenuation from 1.4 to 4 dB/m (at 25°C and 80% humidity). If clutter is present 0.5 m behind the prey, the prey/clutter echo-ratio will be 2.6 dB higher at 90 kHz than at 45 kHz, thus increasing prey conspicuousness.

An added advantage of using low intensity echolocation is that it may prevent prey from detecting an approaching bat. The sound pressure reaching the prey will always be higher than the echo returning to the bats ears, but eared insects, such as moths, generally have much higher hearing thresholds than bats (Wenstrup, 1984; Schmidt et al., 1992; Esser and Daucher, 1996; Koay et al., 1997; Surlykke et al., 1999). While intensity at the insect increases by 20 × log_10_(R) as the bat approaches, the echo the bat receives increases by 40 × log_10_(R). Thus, every time the bat halves the distance to the prey, the sound pressure increases with 6 dB at the prey and 12 dB at the bat. By concurrently reducing its output level by 6 dB, the bat maintains a constant sound pressure at the prey, but still increases the returning echoes by 6 dB. This keeps prey out of the loop while increasing echo strength for the approaching bat (Surlykke, 1988). By emitting low intensity calls, the aerial hawking bat, *Barbastellus barbastellus*, can detect its prey before the prey detects the bat, and by reducing its output level during approach it can remain undetected during the pursuit (Goerlitz et al., 2010). The low-intensity calls from *B. barbastellus* do come at a cost; a reduction in output level also reduces the detection distance for the bat, but given that *B. barbastellus* feeds almost exclusively on eared insects, the benefit of not being detected seems to outweigh the cost of operating at short range.

## Directionality

A directional echolocation signal provides bats with a number of advantages over an omni-directional signal: (1) inherent directional information; by focusing sound in the forward direction, returning echoes are likely to originate from that direction, simplifying object localization. (2) A reduction in clutter; when less sound is radiated to the back and sides of the bat, less sound is reflected off objects of little or no interest in these directions, reducing the amount of information the bat has to process. (3) An increase in source level (on-axis intensity); by focusing energy in a narrow cone instead of radiating it in all directions. On the other hand, a highly directional sound beam will also restrict the bats “field of view” which may be a disadvantage in certain situations.

Beam shape is a spatial filter that determines what information is available to the bat and what information is filtered out before echoes return. This may be critical in light of the very short time bats have to make decisions. A typical pursuit often takes less than half a second and the time to process information and make decisions on a call-to-call basis is even shorter, perhaps a few tens of milliseconds. A clear advantage of a highly adapted and dynamic emission (and reception) system is a reduction in processing load on the receiving side i.e., smart sensing over smart processing. This may be one of the adaptations that allow for the very fast reaction times in echolocating bats, subsequently leading to high foraging, and, in turn, evolutionary, success.

**Figure 2:**
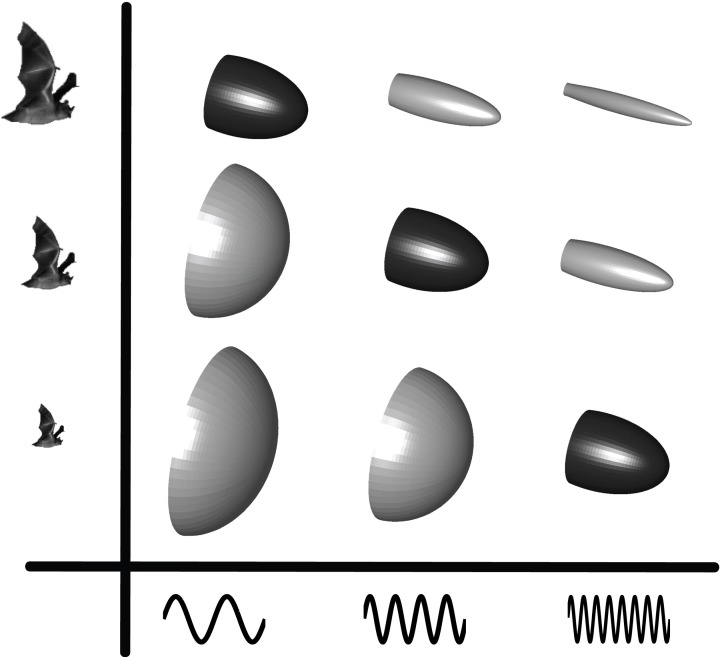
**Echolocation beam shape as a function of emitter size and frequency.** The beam-shape schematics illustrate how directionality increases as either frequency or emitter size increase. The highlighted diagonal beam patterns illustrate how bats of different sizes can converge on similar beam patterns by adjusting the emitted frequency to their size i.e., small bats emit higher frequencies than large bats. Figure from Jakobsen et al. (2013).

Beam shape is determined by the size and shape of the sound emitter and the frequency of the emitted signal (Strother and Mogus, 1970; Urick, 1983) such that an increase in size or frequency generates a more directional sound beam (Figure 2). Frequency is easily measured, but emitter size and shape is not as apparent when dealing with live animals, let alone bats in flight. For bats emitting sound through the open mouth, gape size presumably dictates directionality. Opening the mouth more while emitting a given frequency will generate a more directional beam and vice versa (Surlykke et al., 2009). For nose emitting bats, beam shape is likely dictated by the distance between the nostrils and the size and shape of the nose-leaf (Hartley and Suthers, 1987).

**Figure 3:**
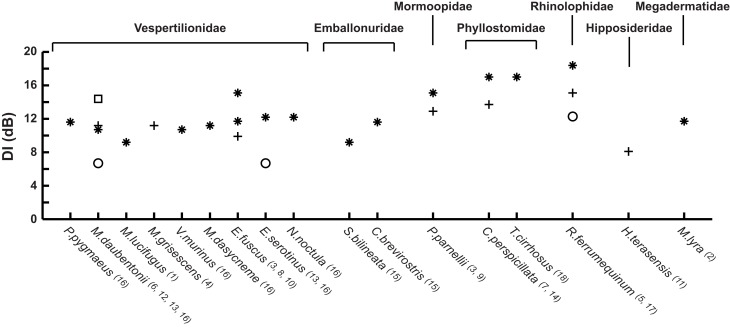
**Directivity index (DI) for the 17 bat species measured to date.** The DI compares the emitted source level with that of an omni-directional source producing a signal with the same acoustic power. The species are grouped by family and arranged within families by increasing size (forearm measurements). Vespertilionids, Emballonurids, and Mormoopids emit sound through the mouth. Phyllostomids, Rhinolophids, Hipposiderids, and Megadermatids emit sound through the nose. ^*^ indicates a recording of a freely flying bat, + indicates that the bat was restrained, □ indicates a field recording, and ○ are recordings of the terminal buzz. Measurements from: ^1^Griffin, 1958; ^2^Möhres and Neuweiler, 1966; ^3^Simmons, 1969; ^4^Shimozawa et al., 1974; ^5^Schnitzler and Grinnell, 1977; ^6^Mogensen and Møhl, 1979; ^7^Hartley and Suthers, 1987; ^8^^1989^; ^9^Henze and O'Neill, 1991; ^10^Ghose and Moss, 2003; ^11^Hiryu et al., 2006; ^12^Surlykke et al., 2009; ^13^Jakobsen and Surlykke, 2010; ^14^Brinkløv et al., 2011; ^15^Jakobsen et al., 2012; ^16^Jakobsen et al., 2013; ^17^Matsuta et al., 2013; ^18^Surlykke et al., submitted. Where measurements were not available from the literature, DI was calculated for a piston source emitting a sound beam with the reported half amplitude angle (the angle where the sound pressure is reduced by 6 dB relative to 0°).

Beam directionality has so far been measured in 17 bat species from seven different families (Figure 3). The methods differ substantially. Many studies were performed on restrained bats, often with calls elicited through stimulation of the brain by implanted electrodes (Shimozawa et al., 1974; Schnitzler and Grinnell, 1977; Mogensen and Møhl, 1979; Hartley and Suthers, 1987, 1989; Henze and O'Neill, 1991; Hiryu et al., 2006). This made it easy to control the bat's position and acoustic axis, but probably prevented active beam shape control by the bats, and in some cases produced calls very different from those produced by freely behaving bats. A few early and most later studies focus mostly on freely behaving bats in the lab (Griffin, 1958; Simmons, 1969; Ghose and Moss, 2003; Jakobsen and Surlykke, 2010; Jakobsen et al., 2012, 2013) with one report from the field (Surlykke et al., 2009). In spite of the large differences in methodology, a few general trends emerge from the data set. It is clear that all bats recorded emit directional signals and it is also clear that they emit a bilaterally symmetrical sound beam. Most results come from the Vespertilionidae (nine species), where directionality is remarkably uniform across species for bats echolocating under similar conditions, in spite of large differences in bat size and emitted frequency (Figure 3). This indicates that directionality may have been one of the major constraints on the evolution of echolocation frequency, forcing small bats to echolocate at higher frequencies to produce a sufficiently directional beam (Jakobsen et al., 2013). Echolocation frequency is also important for echo reflection (Møhl, 1988; Pye, 1993) and ranging accuracy (Stamper et al., 2009) and frequency-dependent directionality may help bats segregate target and clutter echoes (Bates et al., 2011). Thus, echolocation frequency is probably under several simultaneous evolutionary constraints. There is a negative correlation between size and echolocation frequency in most families of bats (Jones, 1999), and it will be interesting to see if this translates into a convergence of beam width for other families than the Vespertilionidae. Data from the emballonurids seem to deviate from this pattern. *Cormura brevirostris* emits a narrower beam in the flight cage than does *Saccopteryx bilineata* (*DI* of 11.5 and 9.3 dB, respectively, Figure 3), but given that emission patterns from only two species have been recorded so far (Jakobsen et al., 2012), conclusive evidence is still lacking. In contrast to other bats Phyllostomids do not appear to show correlation between body size and emitted frequency. Curiously, data show that nose-leaf size is not correlated with body size either in phyllostomids (Hartley and Suthers, 1987; Jones, 1999). Even though the nose-leaf is not the emitter *per se*, it has been shown to define the vertical directionality (Hartley et al., 1989; Vanderelst et al., 2012). Thus, if directionality is a driving force for echolocation frequency in phyllostomids as well, we would expect to find that the emitted frequency is correlated to the size of the nose-leaf and the nostril separation but not to body size.

Bats adapt many features of their echolocation calls in response to changes in their surroundings and to behavioral context. Signal bandwidth and peak frequency are increased in many species when they navigate in cluttered space probably to improve resolution and localization accuracy (Kalko and Schnitzler, 1993). Further, an increase in frequency will result in an increase in directionality. However, as directionality depends on both frequency and effective emitter size, combined changes of the two may result in the opposite effect. This seems to be the case for the vespertilionid *Myotis daubentonii*. When navigating in the field, *M. daubentonii* emits calls with lower peak frequency than in the lab (45 vs. 55 kHz), which by itself would produce a *less* directional beam. Yet, the signals are more directional in the field than in the lab, presumably because the bats also increase their emitter size by opening the mouth wider (Surlykke et al., 2009).

**Figure 4:**
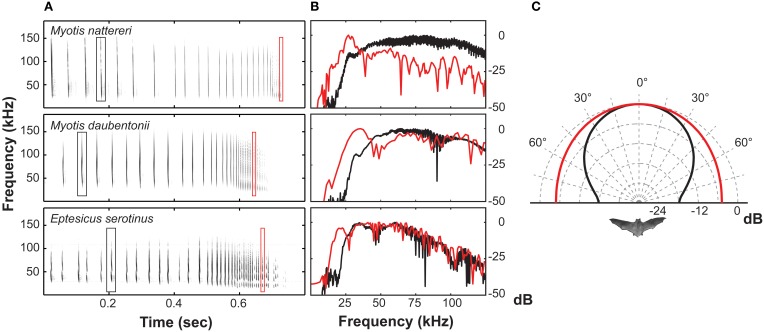
**(A)** Spectrograms of three prey-capture sequences from vespertilionid bats. **(B)** Power spectra illustrating the frequency drop from the approach phase (black trace) to the terminal phase (red trace). **(C)** Measured directionality for approach signals at 55 kHz (black trace) and for buzz II signals at 27.5 kHz (red trace) for *Myotis daubentonii*.

Echolocating bats must adjust directionality not only to adapt to the environment, but also in response to rapid changes in the perceived echo-scene, especially when hunting prey doing their best to escape. At least six orders of insects have ultrasound sensitive ears and exhibit “anti-bat tactics” i.e., they perform erratic escape behaviors like power dives and passive falls in response to intense ultrasound (Miller and Surlykke, 2001). The relatively high directionality of echolocation signals will, in close proximity to prey, become a disadvantage to the bat since the prey only has to move a short distance to escape the bat's sound beam. *M. daubentonii* and *Eptesicus serotinus* (Vespertilionidae) in fact broaden their beam in the last part of prey pursuit by lowering call frequency by roughly an octave (Jakobsen and Surlykke, 2010). A similar frequency drop is seen in a large number of insectivorous vespertilionids and is known as Buzz II (Figure 4). Buzz II calls have been thought an artifact of the extremely high repetition rate of calls emitted during this stage of pursuit, sometimes exceeding 200 calls/s (Kalko and Schnitzler, 1989; Faure and Barclay, 1994). From a purely physiological perspective, however, this seems unlikely as such fast call rates would result in additive tension build-up in the laryngeal muscles, ultimately increasing, rather than reducing call frequency during the buzz (Ratcliffe et al., 2013). Further, many species of echolocating bats use repetition rates as high as vespertilionids but without the frequency drop (Surlykke et al., 1993; Ibáñez et al., 2002). Thus, we argue that the lower frequency of Buzz II calls is not a non-functional epiphenomenon. Rather, it is an adaptive feature that broadens the echolocation beam considerably in the last phase of pursuit to counter the evasive maneuvers performed by many eared insects when exposed to intense ultrasound (Jakobsen and Surlykke, 2010). This argument is corroborated by recent results from horseshoe bats. During prey pursuit, the Japanese greater horseshoe bat, *Rhinolophus ferrumequinum nippon*, will likewise broaden its echolocation beam, but only when the prey moves (Matsuta et al., 2013). In horseshoe bats beam broadening is, contrary to vespertilionids, not achieved by lowering the call frequency. The mechanism underlying the change in beam shape is still unknown, but it is likely facilitated by manipulating the fine structures of the nose leaf (Feng et al., 2012).

As discussed above, the optimal directionality is likely to differ from situation to situation, but also between bats with different feeding ecology. Gleaning bats, foraging in dense vegetation for inconspicuous stationary food items, presumably benefit more from a narrower beam shape than an open space aerial hawking bat. Directionality data from the frugivorous bat, *C. perspicillata*, and the frog-eating bat, *Trachops cirrhosus*, corroborate this. Both phyllostomid species have echolocation beam widths with *DI* of 17 dB (half amplitude angle of ~15°) when flying in a flight cage (Brinkløv et al., 2011, Surlykke et al., submitted). This is considerably narrower than the 10–12 dB (half amplitude angle of ~37°) measured for aerial hawking bats flying in similar conditions (Ghose and Moss, 2003; Jakobsen et al., 2013) (Figure 3). So far the only report of active beam shape adjustments in nose-emitting bats come from the horseshoe bats (Matsuta et al., 2013), an adjustment likely facilitated by manipulating the shape of the nose leaf. However, the phyllostomid, *M. macrophyllum*, shifts maximum energy to the second harmonic in the open, but to the third (or fourth) harmonic in cluttered space (Brinkløv et al., 2010), indicating that phyllostomid bats may also alter beam shape by changing emitted frequency.

## Modeling emission patterns

Modeling bats as physical sound emitters allows predictions about parameters, which cannot or have not been measured. The simple piston model describes the beam pattern of a rigid circular piston oscillating in an infinite baffle and has been used as a model for the emission pattern of mouth emitting bats (Strother and Mogus, 1970; Mogensen and Møhl, 1979):

$$R_{P}(\theta )=\left|\frac{2\times J_{1}(k\times a\times \sin(\theta ))}{k\times a\times \sin(\theta )}\right|$$

*R*_*p*_(θ) is the ratio between the on-axis pressure and the pressure at an angle θ, *J*_1_ is a first order Bessel function of the first kind, *k*, the wavenumber = 2π/λ, λ the wavelength, and *a* is the radius of the piston. Even though, there are a number of obvious differences between bats and the model, the model performs surprisingly well in predicting the emission pattern from mouth emitting bats (Mogensen and Møhl, 1979; Hartley and Suthers, 1989; Jakobsen and Surlykke, 2010; Jakobsen et al., 2012).

The emission pattern from nose emitting phyllostomid bats has been modeled using a two point-source model, but with limited success in particular for freely flying bats (Strother and Mogus, 1970; Hartley and Suthers, 1987; Brinkløv et al., 2011). A model simulating two small pistons with the same separation as the nostrils appears a much better approximation to the horizontal directionality of phyllostomid bats (Vanderelst et al., 2010), but the vertical pattern has so far not been successfully modeled by simple means (see Zhuang and Müller, 2006, 2007; Vanderelst et al., 2010, 2012 for more advanced procedures).

**Figure 5:**
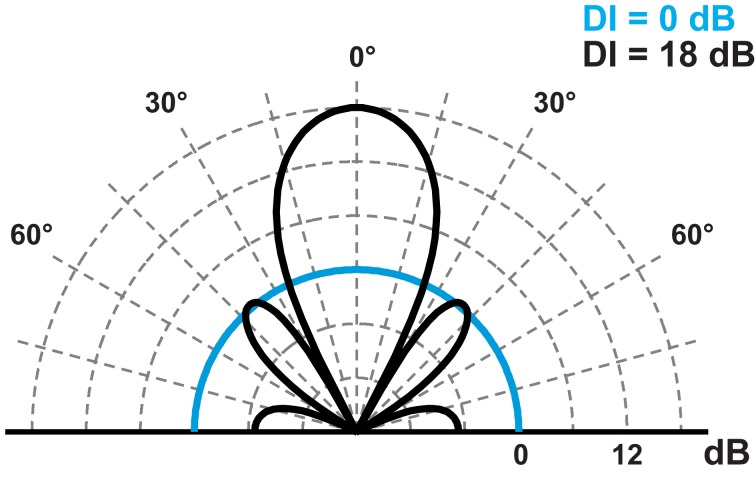
**Beam pattern from an omnidirectional sound source (*DI* = 0 dB, cyan trace) and a directional source following the piston model (*DI* = 18, black trace).** The sources radiate sound of equal acoustic power.

The directionality and intensity of sound signals are not independent features. Intensity changes with directionality, such that an increase in directionality will lead to a corresponding increase in intensity along the acoustic axis. The directivity index (DI) of the source reflects this relationship. A *DI* of e.g., 18 dB implies that sound intensity along the acoustic axis is 18 dB higher than it would be for an omnidirectional sound source radiating sound with the same acoustic power (Figure 5). For the piston model, the DI simply follows from the relation between size and wavelength:

$$DI=20\log_{10}(k\times a)$$

where *k* = 2π/λ, λ is the wavelength, and *a* is the radius of the piston. Using measured data, the calculation of DI is slightly less simple. It requires an estimation of the sound field behind the bat and assumes that the beam is rotationally symmetric (Møhl et al., 2003).

Hence, by increasing or decreasing the directionality of the signal, the on-axis intensity inherently changes as well. *M. daubentonii* emits a source level of 111 dB SPL in the lab and 119 dB SPL in the field. Since the call is broader in the lab (*DI* = 11 dB) than in the field (*DI* = 16 dB) it follows that the bat only increases its output intensity by 3 dB while the remaining 5 dB are accounted for by the greater directionality in the field (Surlykke et al., 2009).

The increase in on-axis intensity with increasing directionality also means that increasing the signal frequency does not necessarily lead to a reduction in detection distance, in spite of the increased atmospheric attenuation at higher frequencies. This is because an increase in frequency increases the signal directionality and thereby the on-axis sound level. Again the situation is simple for the piston model, where a change in frequency from *f*_1_ to *f*_2_ leads to a change in DI of:

$$\Delta DI=20\log_{10}\left(\frac{f_{2}}{f_{1}}\right)$$

**Figure 6:**
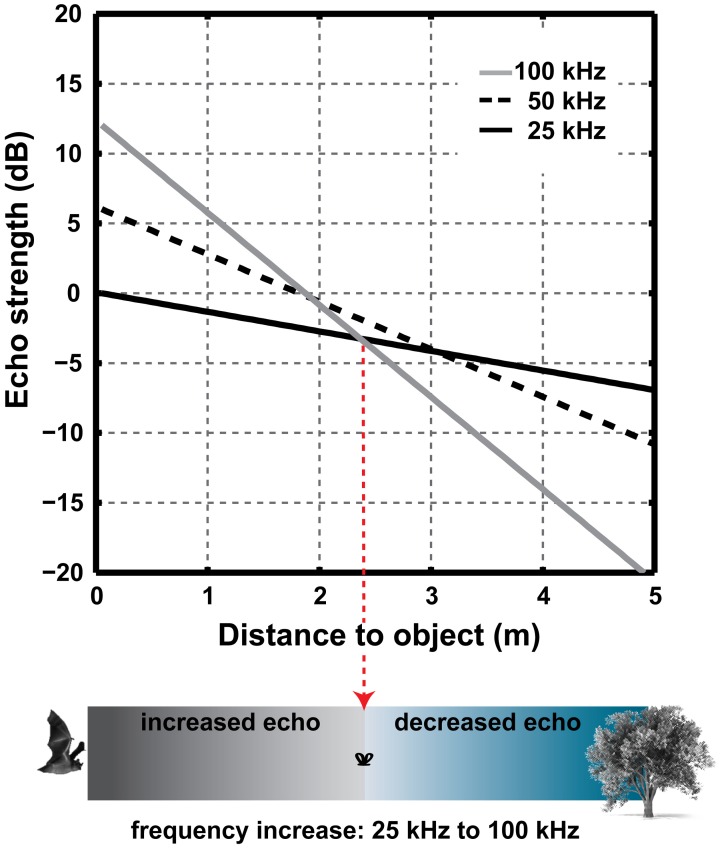
**The combined effect of increased DI and atmospheric attenuation over distance when the emitted frequency is increased from 25 to 50 to 100 kHz and emitter size is constant.** An increase in frequency will lead to an increase in source level and thus in echo strength at short ranges. Note that calculations do not include spherical spreading loss or scattering from the target.

The total atmospheric attenuation depends on the distance the sound travels, whereas, an increase in on-axis sound level affects the source level and thus echo level irrespective of distance. Thus, at short echolocation ranges bats can increase frequency to achieve a higher directionality without sacrificing sonar range. At longer ranges the increase in atmospheric attenuation outweighs the increase in source level. For example a doubling in frequency from 25 to 50 kHz, increases DI and thus source level by 6 dB while the atmospheric attenuation increases from 0.7 to 1.7 dB/m (at 20°C and 50% humidity). Thus, up to a distance of 3 m (two-way travel distance: 6 m), the increased atmospheric attenuation at 50 kHz does not outweigh the increase in source-level. Due to the non-linear increase in atmospheric attenuation with frequency in air, the distance where these two effects cancel each other out depends on the absolute frequencies (Figure 6).

**Figure 7:**
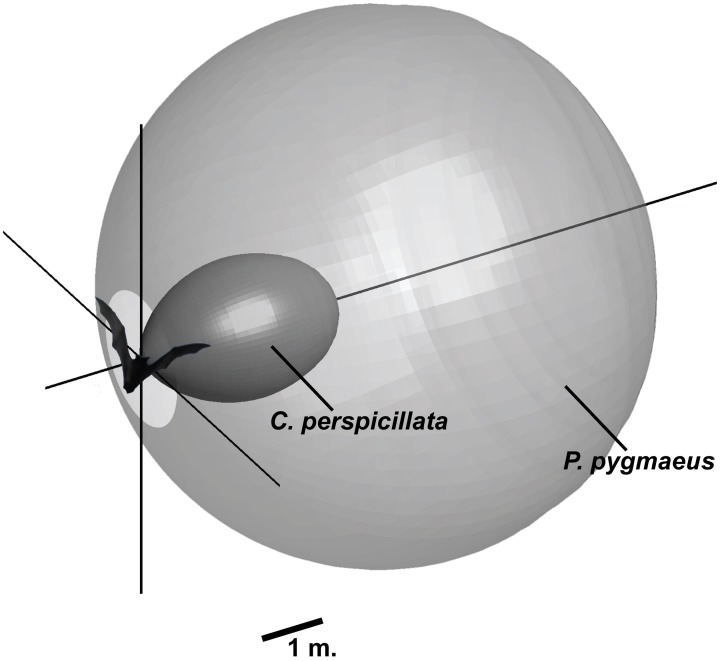
**Search volume for *P. pygmaeus* and *C. perspicillata* navigating an indoor flight room.** The shape illustrates the volume in space where a smooth large surface that absorbs 20 dB of the incoming sound is detectable. Calculations are based on measured parameters (*P. pygmaeus*: 111 dB SPL, 55 kHz, *DI* = 12 dB, *C. perspicillata*: 99 dB SPL, 90 kHz, *DI* = 17). For both bats, the hearing threshold is assumed to be noise limited to 20 dB SPL. The calculations do not account for directional properties on the receiving side.

Many factors combine to define the optimal echolocation signal for a given situation. There are clear differences between bats with different feeding ecologies even when they navigate similar scenarios, indicating a critical role of feeding ecology for how evolution has shaped echolocation signals. Many phyllostomids, such as *C. perspicillata*, feed primarily on fruit and must navigate dense vegetation. *Pipistrellus pygmaeus* is an example of a typical vespertilionid bat hunting moving insects in open fields or along forest edges. The requirements to the sonar systems of these two bats are very different and reflected in the combination of emitted intensity, directionality, and frequency, even when the bats are flying under similar conditions. In the lab, *P. pygmaeus* emits calls at 111 dB SPL with a *DI* of 12 at 55 kHz (peak frequency). Under similar conditions, *C. perspicillata* emits 99 dB SPL, with a *DI* of 17 dB and a peak frequency of 90 kHz. Figure 7 shows the acoustic field of view for the two species and illustrates that the combined effects of intensity, directionality, and frequency generate dramatically different search volume for the two bats. The aerial hawking *P. pygmaeus* uses a sonar signal of much longer range and broader width than the gleaning *C. perspicillata*. *P. pygmaeus* searching for moving insect prey can probably “afford” to scan a relatively large volume of space with each call because its quarry will move in the foreground and thus stand out. *C. perspicillata*, on the other hand, must detect an inconspicuous (motionless) fruit-target in heavy clutter. By reducing the ensonified search volume it probably also focuses its attention on a smaller area and thereby increases the likelihood of detecting desirable objects caught in the sonar beam (Dukas, 2004).

## Conclusion

The combined research on intensity and directionality of echolocation calls from bats show clear differences between restrained and unrestrained bats, and between bats flying in the lab and in the field. These differences highlight the huge flexibility of the echolocation system and highlight the importance of active motor control for perception through echolocation. At the same time, they point to the importance of recording naturally behaving bats in the wild.

The volume of space a bat probes with its echolocation beam is a product of the emitted frequency, intensity, directionality, and call duration. The combined effect of adjustments to these components can result in dramatic changes in the overall search volume. The dynamic control of all acoustic features probably plays a key role in the flexibility and adaptability of bat echolocation and is thus a major contributor to their extreme evolutionary success across a vast range of habitats worldwide. This emphasizes the importance of determining all acoustic features, not just frequency and time parameters, to understand the function of echolocation and its adaptation through evolution to habitats and behavioral contexts.

While our knowledge of both intensity and directionality has increased substantially over recent years, it is clear that we are still barely scratching the surface. Presently, we have directionality measurements from only 17 out of more than 1000 species of echolocating bats. So what appears a general rule today may yet prove to be the exception.

### Conflict of interest statement

The authors declare that the research was conducted in the absence of any commercial or financial relationships that could be construed as a potential conflict of interest.
